# Inhibition of *Clostridioides difficile* toxins TcdA and TcdB by the amiodarone derivative dronedarone

**DOI:** 10.1007/s00210-024-03248-8

**Published:** 2024-06-27

**Authors:** Jauheni Matylitsky, Anica Krieg, Judith Schumacher, Joscha Borho, Holger Barth, Panagiotis Papatheodorou

**Affiliations:** Institute of Experimental and Clinical Pharmacology, Toxicology and Pharmacology of Natural Products, Ulm University Medical Center, Albert-Einstein-Allee 11, 89081 Ulm, Germany

**Keywords:** *Clostridioides difficile* infection, Drug repositioning, Drug repurposing, Bacterial toxin, Toxin inhibitor

## Abstract

The dreaded nosocomial pathogen *Clostridioides difficile* causes diarrhea and severe inflammation of the colon, especially after the use of certain antibiotics. The bacterium releases two deleterious toxins, TcdA and TcdB, into the gut, which are mainly responsible for the symptoms of *C. difficile*-associated diseases (CDADs). Both toxins are capable of entering independently into various host cells, e.g., intestinal epithelial cells, where they mono-O-glucosylate and inactivate Rho and/or Ras GTPases, important molecular switches for various cellular functions. We have shown recently that the cellular uptake of the *Clostridioides difficile* toxins TcdA and TcdB (TcdA/B) is inhibited by the licensed class III antiarrhythmic drug amiodarone (Schumacher et al. in Gut Microbes 15(2):2256695, 2023). Mechanistically, amiodarone delays the cellular uptake of both toxins into target cells most likely by lowering membrane cholesterol levels and by interfering with membrane insertion and/or pore formation of TcdA/B. However, serious side effects, such as thyroid dysfunction and severe pulmonary fibrosis, limit the clinical use of amiodarone in patients with *C. difficile* infection (CDI). For that reason, we aimed to test whether dronedarone, an amiodarone derivative with a more favorable side effect profile, is also capable of inhibiting TcdA/B. To this end, we tested in vitro with various methods the impact of dronedarone on the intoxication of Vero and CaCo-2 cells with TcdA/B. Importantly, preincubation of both cell lines with dronedarone for 1 h at concentrations in the low micromolar range rendered the cells less sensitive toward TcdA/B-induced Rac1 glucosylation, collapse of the actin cytoskeleton, cell rounding, and cytopathic effects, respectively. Our study points toward the possibility of repurposing the already approved drug dronedarone as the preferable safer-to-use alternative to amiodarone for inhibiting TcdA/B in the (supportive) therapy of CDADs.

**Supplementary Information:**

The online version contains supplementary material available at 10.1007/s00210-024-03248-8.

## Introduction

Infections with the (nosocomial) human gut pathogen *Clostridioides difficile* (*C. difficile*) are associated with enteric diseases, such as antibiotics-associated diarrhea and the life-threatening pseudomembranous colitis. The clinical symptoms of such *C. difficile*-associated diseases (CDADs) are clearly related to the action of two protein toxins of the pathogen, namely TcdA (toxin A) and TcdB (toxin B). Both toxins are structurally and functionally highly homologous to each other and act independently on target cells in the human gut (Kelly and LaMont 2008; Aktories et al. 2017; Chandrasekaran and Lacy 2017).

TcdA and TcdB (shortly TcdA/B) belong to the family of clostridial glucosylating toxins (CGTs). In particular, TcdA/B inactive Rho and/or Ras proteins in the host cell cytosol by mono-O-glucosylation. Rho family members are crucial regulators of the actin cytoskeleton, and their inactivation by TcdA/TcdB leads to the breakdown of the actin meshwork. Consequently, cytopathic cell rounding is a typical morphological feature of TcdA/B-intoxicated cultured cells growing in monolayers (Hall 1994; Just et al. 1995; Aktories and Just 2005).

TcdA and TcdB are single-chain toxins with a successive series of functional domains. Both toxins enter into host cells via receptor-mediated endocytosis, depending either on clathrin (TcdB) and/or PACSIN2 (TcdA) (Papatheodorou et al. 2010; Gerhard et al. 2013; Chandrasekaran et al. 2016). To date, several specific host entry receptors for TcdA or TcdB have been identified that interact with different parts of the toxins, either with a C-terminal domain called CROP (combined repetitive oligopeptides) or with additional receptor binding modules upstream of the CROP domain (Gerhard 2017; Papatheodorou et al. 2024). After the acidification of the endocytic vesicles by vesicular adenosine triphosphatases (vATPases), the middle part of the toxins, the so-called translocation domain (TD), changes its structure and inserts itself into the endosomal membrane (Barth et al. 2001; Orrell et al. 2017). As a result, the TD forms a pore in the endosomal membrane, which allows the N-terminal glucosyltransferase domain (GTD) and the adjacent cysteine protease domain (CPD) of the toxins to be translocated into the cytosol (Jank and Aktories 2008). Finally, the CPD is activated by binding of the cytosolic molecule inositol hexakisphosphate (InsP6), followed by autocatalytic cleavage and release of the GTD into the cytosol (Giesemann et al. 2008; Egerer et al. 2009).

Insertion and pore formation of TcdA/B depends on the presence of cholesterol in endosomal membranes. For that reason, compounds that interfere with cholesterol biosynthesis, such as simvastatin, 25-hydroxycholesterol, PF-429242, and U18666A, represent potent pharmacological inhibitors of both toxins (Papatheodorou et al. 2019, 2021). It was shown recently that the licensed drug amiodarone, a multichannel blocker for the treatment of cardiac dysrhythmias (Kodama et al. 1997), is also capable of lowering cholesterol biosynthesis in human cell lines by direct inhibition of the 24-dehydrocholesterol reductase (DHCR24) (Allen et al. 2020; Simonen et al. 2020). Prompted by these findings, we recently demonstrated with cultured human cells and human intestinal organoids that amiodarone exerts a protective effect against TcdA/B, however most likely by direct interference with membrane insertion and/or pore formation of the toxins (Schumacher et al. 2023).

However, amiodarone has serious side effects, including thyroid dysfunction and severe pulmonary fibrosis (Sobol and Rakita 1982; Harris et al. 1983), which may limit the prospect of its use in the treatment of CDI patients. Influence on the thyroid function can be explained due to amiodarone’s chemical similarity to thyroxine and the presence of iodine (Medić et al. 2022). The amiodarone derivative dronedarone represents a new antiarrhythmic drug with a more favorable side effect profile, recently approved for clinical use. Dronedarone is lacking iodine moieties and therefore lacks amiodarone’s organ toxicity. Moreover, dronedarone’s shorter half-life facilitates faster clearance, thereby minimizing the potential for accumulation of toxic concentrations (Yalta et al. 2009; Kozlowski et al. 2012; Khan et al. 2017). However, it is interesting to note that dronedarone is less effective than amiodarone for the maintenance of sinus rhythm (Piccini et al. 2009).

Given the obvious medical advantages of dronedarone over amiodarone particularly regarding side effects, it was now necessary to test whether dronedarone is also able to inhibit TcdA/B. To this end, we performed a series of in vitro intoxication experiments with the African green monkey kidney cell line Vero and the physiologically more relevant colon carcinoma cell line CaCo-2 for analyzing the impact of dronedarone on TcdA/B-induced Rac1 glucosylation, collapse of the actin cytoskeleton, cell rounding, and cytopathic effects, respectively. Taken together, we were able to show that TcdA/B intoxication is inhibited in cultured Vero and CaCo-2 cells that were preincubated with dronedarone at concentrations in the low micromolar range. Our study suggests that dronedarone has the potential to be used as a safer-to-use alternative to amiodarone in the (supportive) treatment of severe CDADs.

## Results

In this study, natively purified TcdB from the historical *C. difficile* strain VPI 10463 was used from the start as representative for both glucosylating toxins of *C. difficile*. However, key findings were then confirmed with native TcdA (also deriving from *C. difficile* strain VPI 10463) or with the medically relevant combination of TcdA and TcdB (denoted as TcdA + B).

### TcdA- and TcdB-induced cell rounding is delayed in dronedarone-preincubated Vero cells

Our first aim was to test the potential inhibitory impact of dronedarone on TcdB intoxication in Vero cells, an established in vitro model system for intoxication studies. Initially, it was required to identify the optimal concentration of dronedarone which is well-tolerated by Vero cells. For that purpose, subconfluent Vero cell monolayers were incubated at 37 °C with increasing concentrations of dronedarone, and cell integrity was analyzed microscopically at three time points: 0 h, 4 h, and 24 h (Supplementary Fig. 1). It turned out that 7.5 µM dronedarone is the optimal concentration at which no morphological changes in the cell monolayers can be observed after 24 h in Vero cells. In contrast, with 10 µM dronedarone, an increased number of cells that had lost their cell integrity and were shrunken were observed after 24 h. No obvious morphological changes were observed at all tested dronedarone concentrations after 4 h of incubation.

**Fig. 1:**
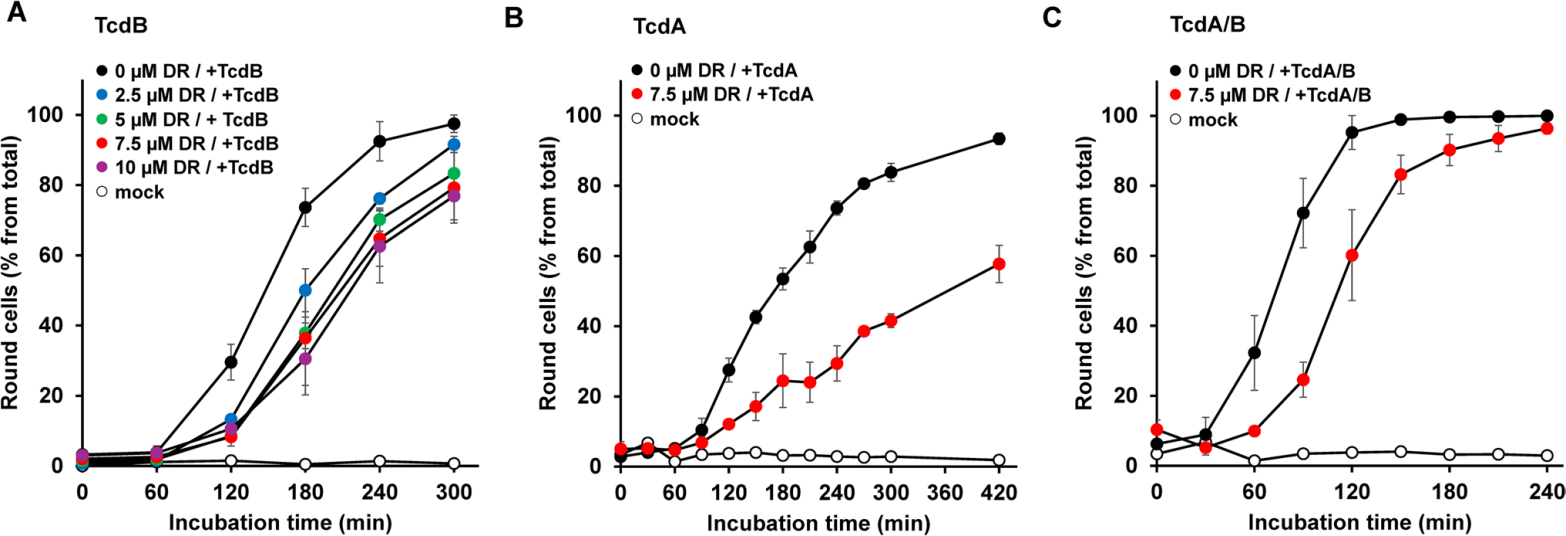
Effect of 1 h dronedarone preincubation on TcdA- and/or TcdB-induced rounding of Vero cells. Vero cells were preincubated with the indicated concentrations of dronedarone (“DR”), followed by the addition of **A** 60 pM TcdB (“ + TcdB”), **B** 180 pM TcdA (“ + TcdA”), or **C** 60 pM TcdB plus 180 pM TcdA (“ + TcdA/B”), and microscopic analysis of the cell morphology. Graphs represent the quantification of toxin-induced cell rounding over time. Shown are the mean percentage values of round cells (in % from total cells), calculated from triplicates (three independent wells). Error bars represent ± SD (standard deviation). “Mock” indicates cells without dronedarone preincubation and without toxin treatment

We then tested whether preincubation of Vero cells for 1 h at 37 °C with increasing concentrations of dronedarone (0 µM, 2.5 µM, 5 µM, 7.5 µM, and 10 µM) delays TcdB intoxication. Following exposure to 60 pM TcdB, cell morphology was assessed microscopically at various time points over 5 h. Representative images from time points 0 and 180 min after addition of TcdB are shown in Supplementary Fig. 2. Interestingly, all tested dronedarone concentrations rendered the Vero cells less sensitive toward TcdB, except for 0 µM dronedarone. An hourly quantification of TcdB-induced cell rounding revealed a concentration-dependent delay of intoxication in dronedarone-preincubated cells (e.g., ~ 60% reduction at time point 180 min for 10 µM dronedarone) (Fig. 1A). The time course experiment was then repeated with TcdA (180 pM) and with TcdA/B (180 pM TcdA plus 60 pM TcdB) and with Vero cells preincubated for 1 h at 37 °C with 7.5 µM dronedarone. Importantly, dronedarone was capable of also delaying the intoxication of Vero cells with TcdA (Fig. 1B) and TcdA/B (Fig. 1C).

### Dronedarone preincubation inhibits TcdB-induced Rac1 modification and collapse of the actin cytoskeleton in Vero cells

**Fig. 2:**
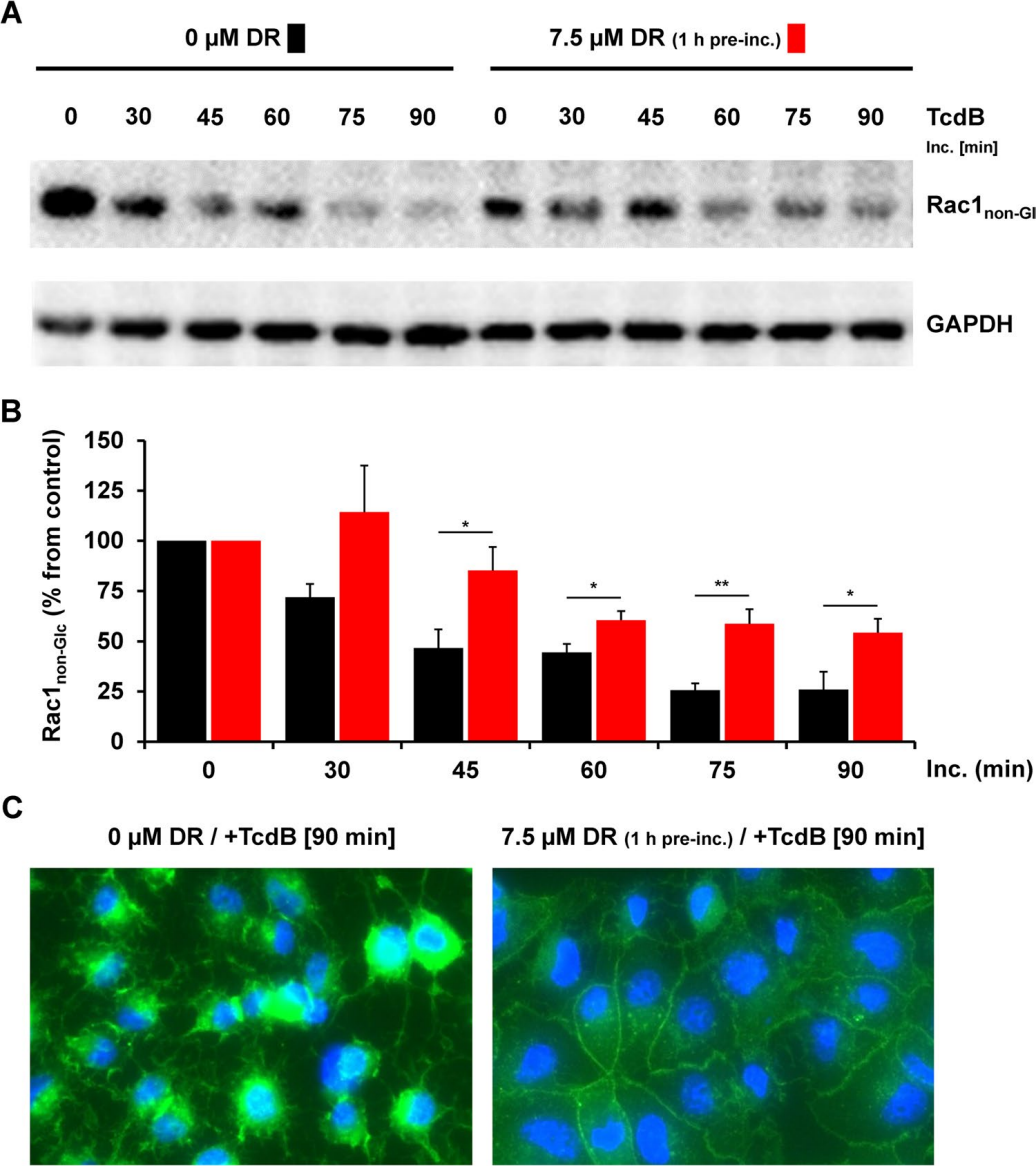
Effect of 1 h dronedarone preincubation on TcdB-induced Rac1 glucosylation and collapse of the actin cytoskeleton in Vero cells. **A**, **B** Following 1 h preincubation without dronedarone (black square, “0 µM DR”) or with 7.5 µM dronedarone (red square, “7.5 µM DR”), Vero cells were treated with 60 pM TcdB and incubated for the indicated time intervals. **A** Immunoblots against non-glucosylated Rac1 (“Rac1_non-Glc_”) and GAPDH (loading control), obtained with whole-cell lysates from toxin-treated Vero cells. **B** Bar chart shows the quantification of the Rac1_non-Glc_ signals (normalized with GAPDH signals) shown in (**A**) over time and relative to the starting time point of intoxication (“0 min”), which was set to 100% for each group of samples (black bars, 0 µm dronedarone; red bars, 7.5 µM dronedarone). Error bars represent ± SEM (standard error of the mean), calculated from five experiments performed in independent wells and on 3 different days. Asterisks indicate statistical significance at each time point between black and red bars with *p < 0.05 and **p < 0.01. **C** Vero cells preincubated for 1 h without (“0 µM DR”; DMSO solvent only) or with 7.5 µM dronedarone (“7.5 µM DR”) were intoxicated for 90 min with 60 pM TcdB (“ + TcdB [90 min]”), followed by fluorescence microscopy after staining of actin filaments with FITC-conjugated phalloidin (green signals) and nuclei with Hoechst 33342 (blue signals). Shown images represent the overlay images of fluorescent actin filaments and nuclei in Vero cells at time point 90 min after TcdB intoxication

Next, we performed more specific experiments to confirm the inhibitory effect of dronedarone on TcdB intoxication of Vero cells on a molecular level. To this end, whole-cell lysates from Vero cells that were intoxicated for increasing time intervals (0 to 90 min) with 60 pM TcdB were generated, followed by immunoblotting for the analysis of the glucosylation status of the TcdB target substrate Rac1 using an antibody that recognizes only the non-glucosylated form of Rac1 (denoted as Rac1_non-Glc_). Within this experimental setting, a reduction in Rac1_non-Glc_ immunoblot signal directly correlates with TcdB intoxication. Intriguingly, whole-cell lysates from TcdB-exposed Vero cells displayed a time-dependent decrease in Rac1_non-Glc_ signal, which was less pronounced in dronedarone-preincubated cells (Fig. 2A and B). Figure 2A shows a representative immunoblot demonstrating this effect and in Fig. 2B is the relative Rac1_non-Glc_ signal intensity (normalized to GAPDH) quantified over time. Additionally, we investigated the influence of dronedarone preincubation on the dynamics of the actin cytoskeleton in Vero cells after TcdB intoxication using fluorescence microscopy. To this end, F-actin filaments were stained with FITC (fluorescein isothiocyanate)-conjugated phalloidin, after intoxication of the cells for 90 min at 37 °C with 60 pM TcdB. As expected, TcdB triggered actin cytoskeleton collapse in Vero cells without dronedarone preincubation, resulting in cell rounding (Fig. 2C, left image). Conversely, dronedarone-preincubated Vero cells retained F-actin filaments and maintained their normal morphology despite TcdB intoxication (Fig. 2C, right image).

### Dronedarone preincubation delays intoxication of CaCo-2 cells with TcdA and TcdB

We have shown above that preincubation with dronedarone renders Vero cells less sensitive to TcdA and TcdB. To confirm these findings, in a second, pathophysiologically more relevant cell line, we sought out to perform intoxication experiments with the human colon carcinoma cell line CaCo-2. We found in a preliminary experiment that growing CaCo-2 cells exhibited a normal morphology when incubated for 3 h with up to 30 µM dronedarone (Supplementary Fig. 3A). Notably, small inclusion bodies appeared within CaCo-2 cells after incubation for 3 h with 30 µM dronedarone (Supplementary Fig. 3B). Since in the following experiments CaCo-2 cells would be preincubated with dronedarone only for 1 h, we decided to measure by SYTOX-staining if an 1 h incubation with 30 µM dronedarone has any effect on the cell integrity of CaCo-2 cells. By this approach we found that only a minor portion of CaCo-2 cells of about 10% was stained with SYTOX and thus membrane-compromised after incubation for 1 h with 30 µM dronedarone (Supplementary Fig. 4).

**Fig. 3:**
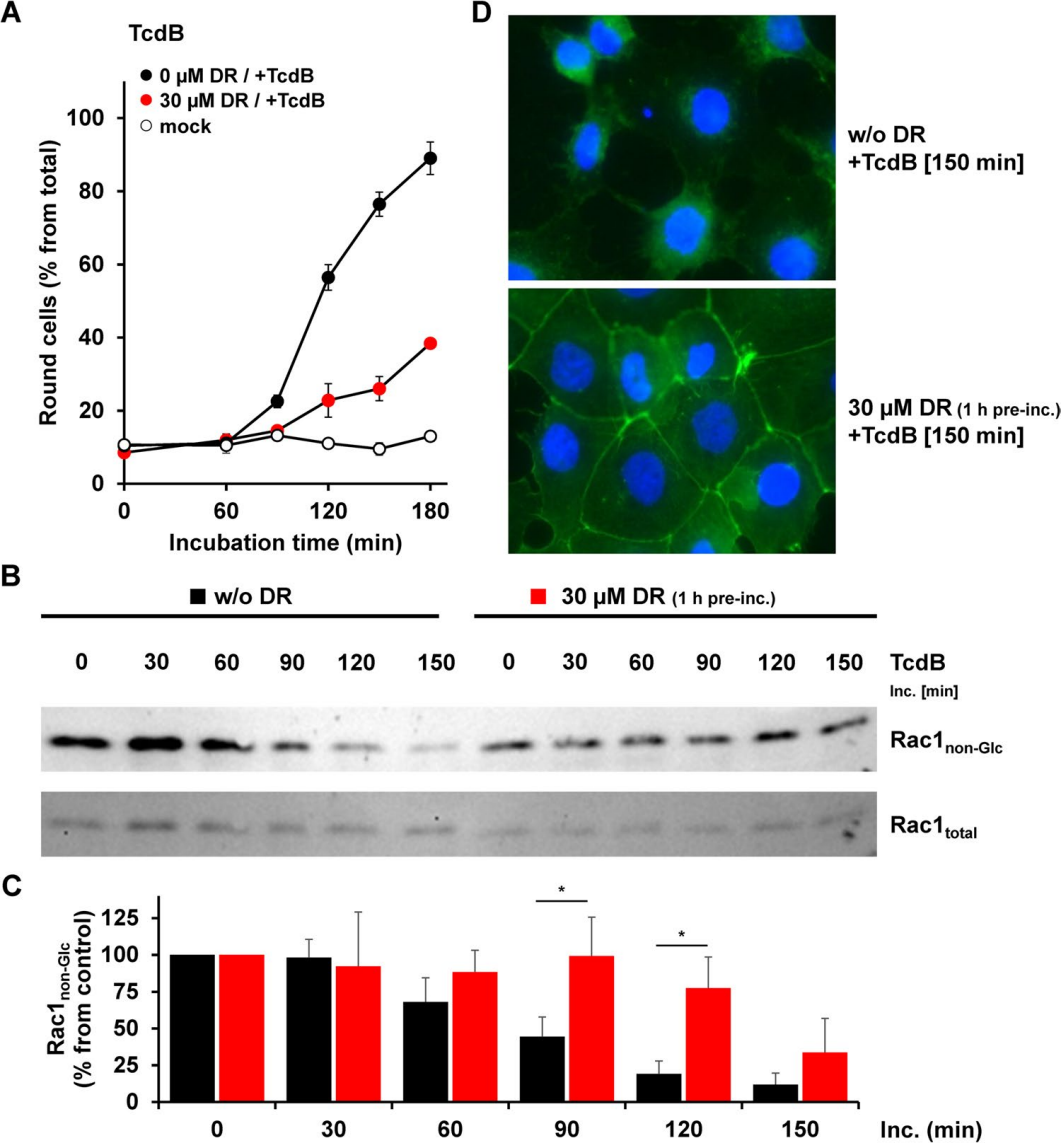
Effect of 1 h dronedarone preincubation on TcdB-induced cell rounding, Rac1 glucosylation, and collapse of the actin cytoskeleton in CaCo-2 cells. **A** CaCo-2 cells were preincubated without dronedarone (“0 µM DR”) or with 30 µM dronedarone (“30 µM DR”), followed by the intoxication with 200 pM TcdB (“ + TcdB”) and microscopic analysis of the cell morphology. Graph represents the quantification of TcdB-induced cell rounding over time and shows the mean percentage values of round cells (in % from total cells), calculated from triplicates (three independent wells). Error bars represent ± SD. “Mock” indicates cells without dronedarone preincubation and without toxin treatment. **B**, **C** Following 1 h preincubation without dronedarone (black square, “0 µM DR”) or 30 µM dronedarone (red square, “30 µM DR”), CaCo-2 cells were treated with 200 pM TcdB and incubated for the indicated time intervals. **B** Immunoblots against non-glucosylated Rac1 (“Rac1_non-Glc_”) and total Rac1 (“Rac1_total_,” loading control) are shown, obtained with whole-cell lysates from TcdB-treated CaCo-2 cells. **C** Bar chart shows the quantification of the Rac1_non-Glc_ signals (normalized with Rac1_total_ signals) shown in (**B**) over time and relative to the starting time point of intoxication (“0 min”), which was set to 100% for each group of samples (black bars, 0 µm dronedarone; red bars, 30 µM dronedarone). Error bars represent ± SD, calculated from three separate experiments performed. Asterisks indicate statistical significance at each time point between black and red bars with *p < 0.05. **D** CaCo-2 cells preincubated for 1 h without (“0 µM DR”) or with 30 µM dronedarone (“30 µM DR”) were intoxicated for 150 min with 200 pM TcdB (“ + TcdB [150 min]”), followed by fluorescence microscopy after staining of actin filaments with FITC-conjugated phalloidin (green signals) and nuclei with Hoechst 33342 (blue signals). Shown images represent the overlay images of fluorescent actin filaments and nuclei in CaCo-2 cells at time point 150 min after TcdB intoxication

At first, we tested with various approaches whether 30 µM dronedarone exhibits a robust inhibitory effect against 200 pM TcdB in CaCo-2 cells. Importantly, we could observe a strong delay in cell rounding induced by TcdB when cells were preincubated for 1 h with 30 µM dronedarone (Fig. 3A). In addition, TcdB-mediated Rac1 glucosylation was also decreased over time in CaCo-2 cells preincubated for 1 h with 30 µM dronedarone (Fig. 3B and C). A representative immunoblot is shown Fig. 3B, and the quantification of the relative Rac1_non-Glc_ signal intensity (normalized to total Rac1) over time is shown in Fig. 3C. Finally, F-actin filaments and normal morphology were still retained in CaCo-2 cells preincubated for 1 h with 30 µM dronedarone upon intoxication with TcdB for 150 min. Interestingly, preincubation of CaCo-2 cells with 7.5 µM dronedarone was also sufficient to render CaCo-2 cells less sensitive to TcdB- and TcdA-induced cell rounding (Supplementary Fig. 5).

Taken together, we were able to prove with various experimental approaches the inhibitory potential of dronedarone against TcdA and TcdB in two different mammalian cell lines. Our study manifests dronedarone as a safer-to-use drug repurposing alternative to amiodarone for the inhibition of TcdA/B in the (supportive) therapy of CDADs.

## Discussion

We found in a recent seminal study that the class III antiarrhythmic drug amiodarone, which is commonly used in the treatment of cardiac dysrhythmias, is a potent inhibitor of the *C. difficile* toxins TcdA and TcdB (Schumacher et al. 2023). As we could show, amiodarone most likely interferes with the translocation pore of both toxins, but additional features of the compound, such as the inhibition of cholesterol biosynthesis (Allen et al. 2020; Simonen et al. 2020) and endosomal acidification (Sanchez et al. 2007), might contribute in inhibiting the toxins’ entry into target cells. Unfortunately, repurposing of amiodarone for the treatment of CDI is hampered by amiodarone’s extracardiac toxicity, including severe side effects, such as thyroid dysfunction and severe pulmonary fibrosis (Sobol and Rakita 1982; Harris et al. 1983). Therefore, the purpose of our current study was to test whether dronedarone, an amiodarone derivative with significantly improved side effect profile, is also capable of inhibiting the *C. difficile* toxins TcdA and TcdB.

With a series of experimental approaches and by using two different mammalian cell lines, including the pathophysiologically relevant human cell line CaCo-2, we could clearly show in vitro that dronedarone inhibits cell intoxication by TcdA and TcdB, but also by the medically relevant combination of both (TcdA plus TcdB). Because dronedarone is capable of inhibiting both toxins, TcdA and TcdB, it is highly unlikely that dronedarone interferes with the interaction of the toxins with cell surface receptors, due to the fact that both toxins engage different receptors for entry into host cells (Papatheodorou et al. 2024). In the current study, we did not further characterize the underlying inhibitory mechanism of dronedarone. However, given to the structural and functional similarity between amiodarone and dronedarone, we assume that both compounds inhibit the *C. difficile* toxins in the same way by interfering with pore formation and/or translocation of TcdA and TcdB.

As already observed in our previous study with amiodarone (Schumacher et al. 2023), in the current study, dronedarone seems (at least in Vero cells) to inhibit TcdA more efficiently than TcdB. The inhibitory effect of amiodarone and dronedarone on TcdA/B as explained above likely involves its interaction with the translocation pore. This interaction could be direct, potentially clogging the pore itself, or indirect, by altering the surrounding lipid environment and hindering pore function. Our findings suggest that the TcdA translocation pore might be either more susceptible to amiodarone and dronedarone binding or more sensitive to changes in its lipid milieu influenced by these drugs. In direct comparison to amiodarone, dronedarone seems to represent a more potent inhibitor against TcdA/B, since an almost 2 to 3 times lower concentration of dronedarone compared to amiodarone was cytoprotective against both toxins. Both dronedarone and amiodarone induce a comparable delay in TcdA/B intoxication; however, both compounds do not result in a complete inhibition. Whereas amiodarone has been shown to inhibit also other toxins besides *C. difficile* TcdA/B, such as lethal and edema toxin from *Bacillus anthracis* and diphtheria toxin from *Corynebacterium diphtheriae* (Sanchez et al. 2007), our study is the first to demonstrate dronedarone’s inhibitory effect on bacterial toxins. Notably, in a parallel study submitted by our laboratory to the same journal issue (Jia et al. 2024), dronedarone was found to inhibit also the intoxication of cells by the pertussis toxin of *Bordetella pertussis*.

Notably, amiodarone and dronedarone share a benzofuran ring linked to a benzoyl group, but amiodarone uniquely possesses two iodide atoms on the benzoyl moiety (Kathofer et al. 2005; Siddiqui et al. 2016). We thus speculate that either only the benzofuran ring or the entire benzoyl benzofuran backbone of both compounds might play as pharmacophore a pivotal role in inhibiting TcdA and TcdB. To test this hypothesis, other natural benzofurans, such as angelicin, xanthotoxin, bergapten, nodekenetin, and usnic acid compounds (Kirilmis et al. 2008), or already approved drugs with a benzofuran unit, such as griseofulvin, ramelteon, vilazodine, citalopram, saprisartan, and benzbromarone (Abbas and Dawood 2023), might be worth to be analyzed for their inhibitory activity against TcdA/B. Several other natural benzofuran compounds have been summarized and are described by Miao and colleagues (Miao et al. 2019). Of interest is also the compound AMIODER, a new benzofuran derivative based on the amiodarone structure with antifungal and antiparasitic activities (Hejchman et al. 2012; Pinto-Martinez et al. 2018; Martinez-Sotillo et al. 2019; de Souza et al. 2022).

Chemically most similar to amiodarone and dronedarone are the so-called aurones, which are flavonoid-type plant compounds with a benzofuran ring coupled to a benzylidene instead of a benzoyl group. Aurones have garnered significant attention for their diverse biological activities, including anti-microbial and anti-cancer properties (Demirayak et al. 2015; Hassan et al. 2018; Alsayari et al. 2019; Abbas and Dawood 2023). Future studies should reveal whether some aurones exhibit an inhibitory activity against TcdA and TcdB.

Drug repurposing of dronedarone might represent a safe antitoxin strategy for the (supportive) therapy of severe *C. difficile*-associated diseases (CDADs). The maximal steady-state plasma concentration of dronedarone ranges between 84 and 167 ng/ml (Iram et al. 2016), corresponding to 0.15 to 0.3 µM. Notably, the inhibitory concentration of dronedarone used in our study was in the lower micromolar range and thus approximately one order of magnitude higher. However, for treating severe CDADs, a rather short-term use and rectal administration of dronedarone at locally higher doses might be conceivable.

## Materials and methods

### Cell culture

Vero cells were cultivated in Minimum Essential Medium (MEM; Fisher Scientific GmbH, Schwerte, Germany; #11524426) supplemented with 10% fetal calf serum (FCS), 1% sodium pyruvate, 1% non-essential amino acids, 1% penicillin/streptomycin, and 2 mM L-glutamine. CaCo-2 cells were grown in Dulbecco’s Modified Eagle’s Medium (DMEM; Fisher Scientific GmbH, Schwerte, Germany; #11594486) supplemented with 10% FCS, 1% sodium pyruvate, and 1% penicillin/streptomycin. Both cell lines were maintained in the incubator at 37 °C and 5% CO_2_ under humidified conditions.

### Toxins and other reagents

TcdA and TcdB from *C. difficile* strain VPI 10463 were generously provided by Klaus Aktories (University of Freiburg, Germany) and purified as described before (Just et al. 1997). Dronedarone hydrochloride (D9696) was ordered from Merck (Darmstadt, Germany), dissolved in DMSO (10 mM stock solution) and stored in aliquots at − 20 °C.

### Cell rounding assay

A cell rounding assay was performed to microscopically assess the intoxication of Vero and/or CaCo-2 cells with TcdA and/or TcdB. To this end, cells were seeded into the wells of a 24-well plate and grown 1 to 2 days until they reached subconfluency. Next, the toxins TcdA and/or TcdB were added at final concentrations as indicated directly into the overlaying growth medium of the cells, followed by further incubation of the well plates at 37 °C in the incubator. For inhibition experiments, dronedarone (solved in DMSO) or solvent control (only DMSO) was added directly into the overlaying growth medium of the cultured cells in the wells and incubated for indicated time intervals at 37 °C, prior to intoxication with TcdA and/or TcdB. Eventually, microscopic images were obtained over time at indicated time points and the number of total and round cells counted manually with the assistance of the Neuralab online tool (https://neuralab.de). Typically, a cell rounding assay was performed with triplicates (three independent wells) for calculating the mean percentage values of cell rounding. Leica DMi1 equipped with a Leica Flexacam C1 camera (Leica, Wetzlar, Germany) was used for analyzing the morphology of cell monolayers.

### Preparation of whole-cell lysates and Rac1 immunoblotting

Whole-cell lysates were generated from cultured cells growing in wells of 24-well plates. After removal of the culture medium, the cell monolayers were optionally frozen at − 20 °C and rethawed or were directly resuspended in 2.5-fold pre-heated (95 °C) Laemmli buffer. Following heat denaturation for 5 to 10 min at 95 °C, cell lysate proteins were separated by SDS-PAGE and transferred by Western blotting onto a nitrocellulose membrane. Primary mouse anti-Rac1 antibody clones 102 (#610651; BD Biosciences, Heidelberg, Germany) and 23A8 (#05–389, Upstate®; Merck, Darmstadt, Germany) were used for the immunodetection of non-glucosylated and total Rac1, respectively. GAPDH was detected with primary mouse anti-GAPDH antibody (sc-365062; G-9; Santa Cruz Biotechnology, Dallas, USA). Horseradish peroxidase (HRP)-coupled goat anti-mouse IgG (H + L) (#31430; Thermo Fisher Scientific, Waltham, USA) was used as secondary antibody. Antibody signals were developed by enhanced chemiluminescence and images were captured with the iBright Imaging System (Invitrogen). The quantification of antibody signals occurred with ImageJ.

### FITC-phalloidin staining of F-actin and fluorescence microscopy

Briefly, Vero or CaCo-2 cells were seeded into the wells of a chambered coverslip with 8 individual wells (µ-Slide 8 Well, ibiTreat, Cat. No. 80826; ibidi GmbH, Gräfelfing, Germany) and grown to subconfluency. Following incubation with inhibitors and/or toxins as indicated, cells were fixed with 4% paraformaldehyde and permeabilized with 0.4% Triton X-100, and the procedure stopped with 100 mM glycine. After blocking with 5% skim milk blocking solution, cells were serially incubated with FITC (fluorescein isothiocyanate)-conjugated phalloidin (P5282; Merck, Darmstadt, Germany) for staining of F-actin and Hoechst 33342 (Thermo Fisher Scientific, Schwerte, Germany) for staining nuclei. The All-in-One Fluorescence Microscope BZ-X800 (Keyence Deutschland GmbH, Neu-Isenburg, Germany), equipped with a Plan Apochromat 40X objective and BZ-X filters for DAPI (OP-87762; detection of Hoechst 33342 signals) and GFP (OP-87763; detection of FITC signals), was used for obtaining fluorescent microscopic images.

### Flow cytometric detection of dead cells with SYTOX green dye

Briefly, CaCo-2 cells were grown in 12-well plates to subconfluency and incubated with inhibitors and/or toxins as indicated. Then, cells were trypsinized, transferred to reaction tubes, and incubated for 15 min at room temperature with SYTOX™ Green Ready Flow™ Reagent (R37168; Thermo Fisher Scientific, Schwerte, Germany), followed by flow cytometric analyses by the FACSCelesta device (BD, Heidelberg, Germany; blue laser 488 nm, 530/30 filter). The open source flow cytometry software Flowing Software (Turku Bioscience Centre) was used for the discrimination and quantification of SYTOX-incorporating and SYTOX-non-incorporating cells via histogram analysis (cell count vs. green fluorescence).

### Statistics

If not otherwise stated, all experiments were performed at least twice and, typically, with at least three independent replicates (e.g., three independent wells) per experiment. Unpaired *t*-test was used for calculating the significance of differences between two groups. Resulting *p*-values were indicated directly in diagrams by asterisks as follows: *p < 0.05, **p < 0.01.

## Conclusion

We have shown in a previous study that the antiarrhythmic drug amiodarone is capable of inhibiting the cell deleterious effects of the toxins TcdA and TcdB from the human gut pathogen *Clostridioides difficile*. In the current follow-up study we were able to show that dronedarone, a less side-effects-prone derivative of amiodarone, is also inhibiting TcdA and TcdB. Our study opens the venue of repurposing dronedarone as safer-to-use alternative to amiodarone in the (supportive) therapy of CDADs.

## Supplementary Information

Below is the link to the electronic supplementary material.

Supplementary file1 (DOCX 2572 KB)

## Data Availability

The data that support the findings of this study are available from the corresponding author, Panagiotis Papatheodorou, upon reasonable request.
