# Supplementary material for: Inhibition of Clostridioides difficile toxins TcdA and TcdB by the amiodarone derivative dronedarone
Source: Naunyn Schmiedebergs Arch Pharmacol. 2024 Jun 27;397(12):9877–85. doi: 10.1007/s00210-024-03248-8 (PMC11582217; doi:10.1007/s00210-024-03248-8)
Supplement: Supplementary file 1 — Supplementary file1 (DOCX 2572 KB) [file 210_2024_3248_MOESM1_ESM.docx]

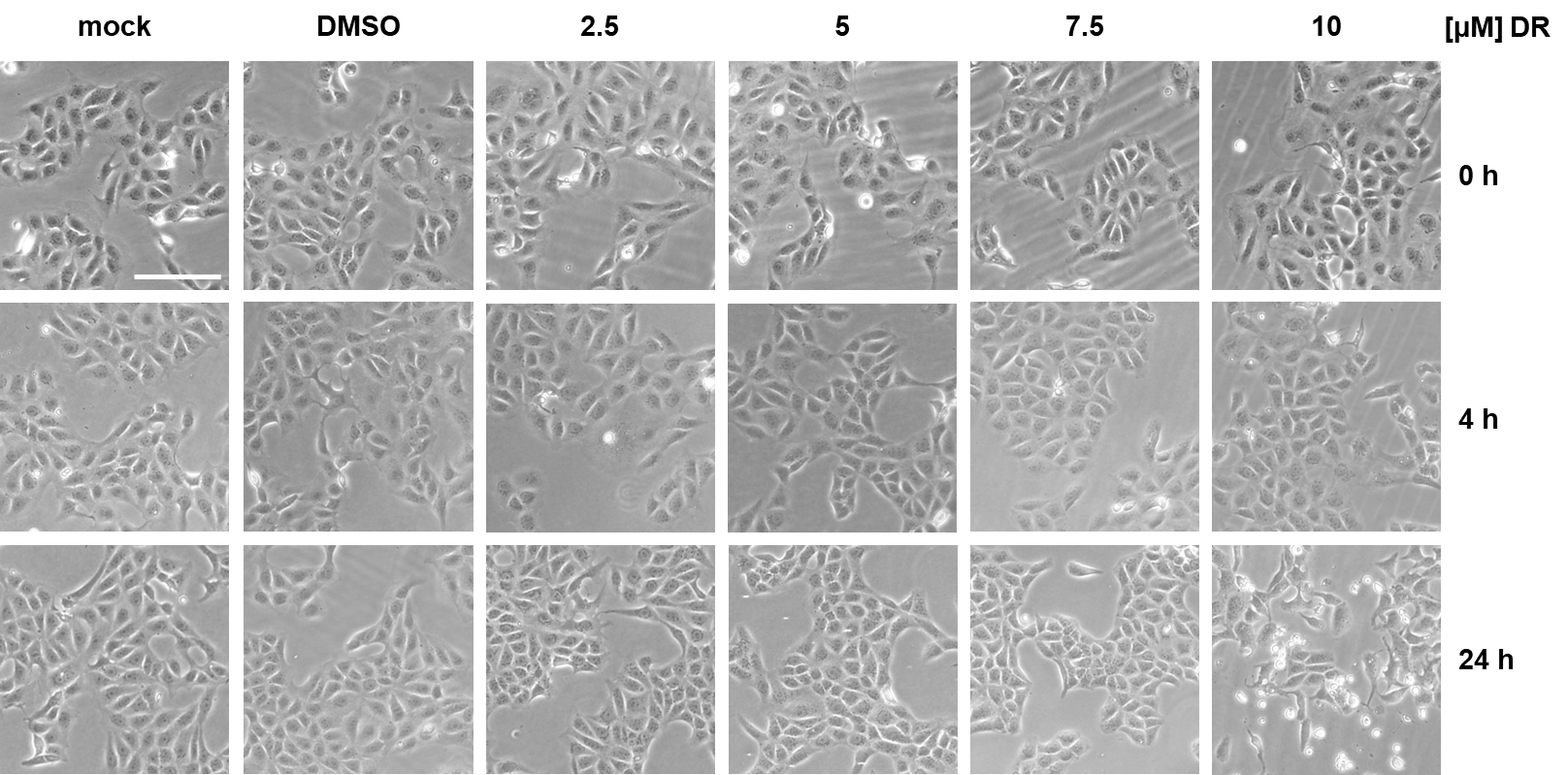


**Supplementary Figure 1**. Effect of dronedarone on Vero cells. Vero cells were incubated with increasing concentrations of dronedarone (‘DR’) or solvent only (‘DMSO’) or were left without treatment (‘mock’), prior to microscopic analysis of the cell morphology and integrity. Shown are the microscopic images of Vero cells after 0, 4 and 24 h of incubation. Shown scale bar represents 100 µm.


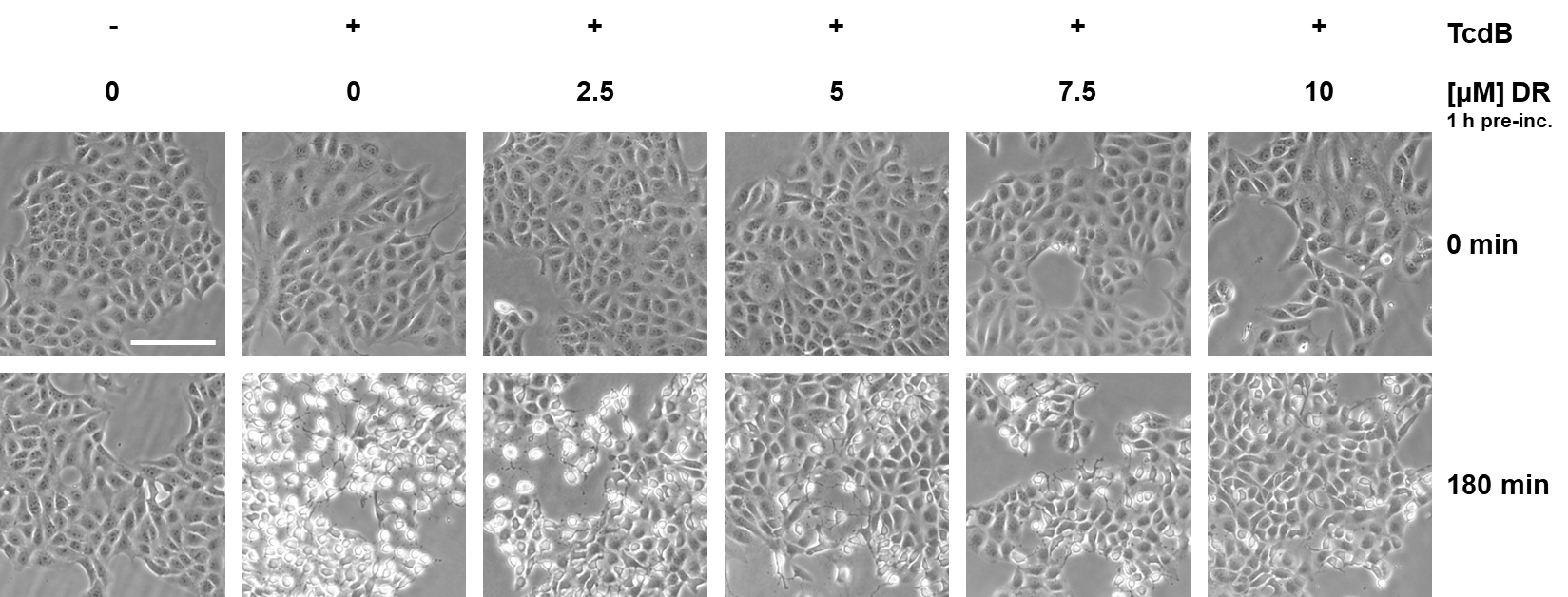


**Supplementary Figure 2**. Effect of 1 h dronedarone preincubation on TcdB-induced rounding of Vero cells. Shown are representative images from time points 0 and 180 min after addition of 60 pM TcdB (‘+TcdB’) to Vero cells preincubated for 1 h with increasing concentrations of dronedarone (‘DR’). Control cells were either preincubated without dronedarone (‘0 µM DR’, ‘+TcdB’) or were left without dronedarone preincubation and toxin treatment (‘0 µM DR’, ‘-TcdB’). The corresponding time course experiment is shown in Figure 1A. Scale bar is 100 µm.


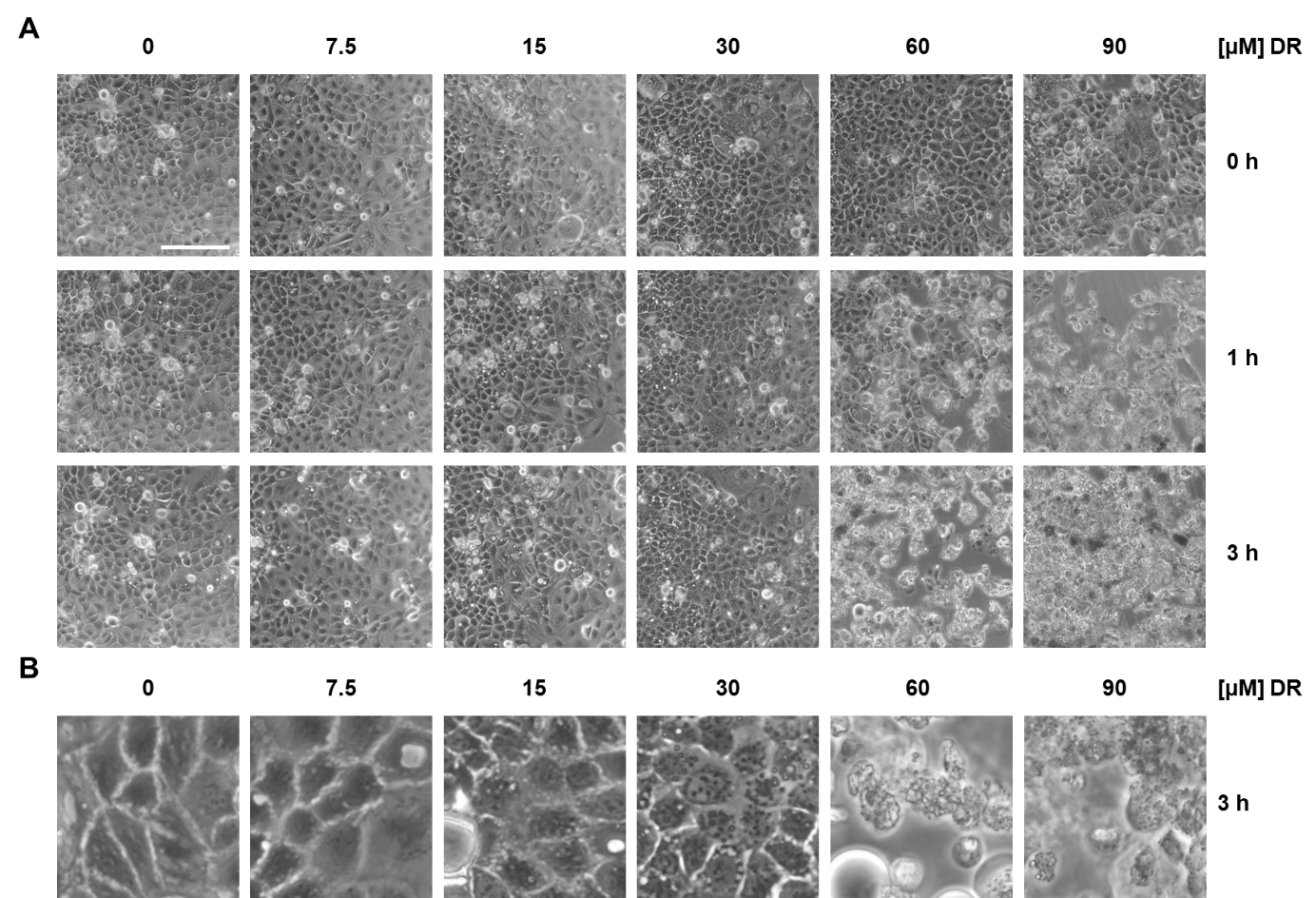


**Supplementary Figure 3**. Effect of dronedarone on CaCo-2 cells. (A) CaCo-2 cells were incubated with increasing concentrations of dronedarone (‘DR’) as indicated, prior to microscopic analysis of the cell morphology and integrity. Shown are the microscopic images of CaCo-2 cells after 0 h, 1 h and 3 h of incubation. Shown scale bar represents 100 µm. (B) Sections from images ‘3h’ shown in (A) in 5-fold magnification.


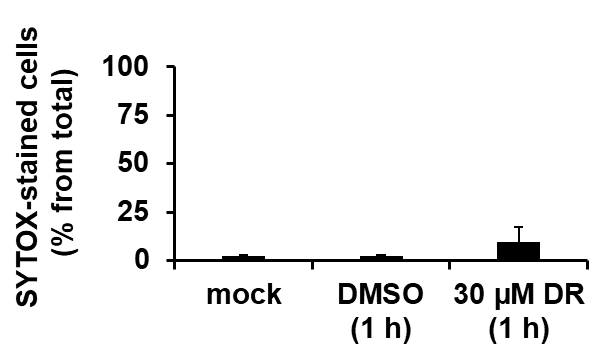


**Supplementary Figure 4**. Effect of dronedarone on CaCo-2 cells. CaCo-2 cell monolayer were incubated for 1 h with 30 μM dronedarone (‘DR’) or only with the same volume of the solvent DMSO (‘DMSO’), following detachment by trypsinization, addition of SYTOX Green dye and measurement of green-fluorescent cells via flow cytometry. Bar graph shows the mean values from the percentage of SYTOX-stained CaCo-2 cells at each condition in % from total cells. ‘Mock’ indicates cells without any treatment. Error bars represent SD, calculated from the three independent experiments, each performed with duplicates.


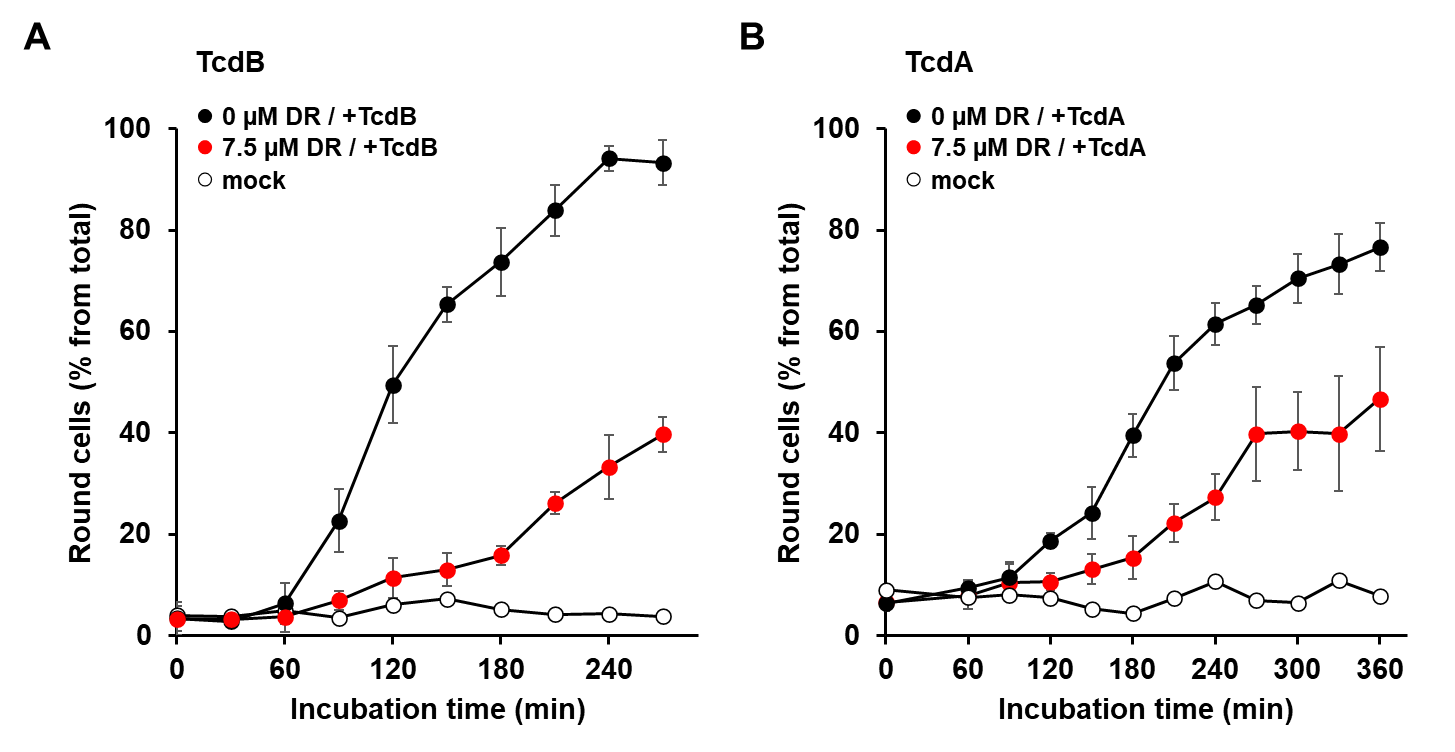


**Supplementary Figure 5**. Effect of 1 h dronedarone preincubation on TcdA- or TcdB-induced cell rounding in CaCo-2 cells. (A,B) CaCo-2 cells were preincubated without dronedarone (‘0 µM DR’) or with 7.5 µM dronedarone (‘7.5 µM DR’), followed by the intoxication with (A) 200 pM TcdB (‘+TcdB’) or (B) 2 nM TcdA (‘+TcdA’) and microscopic analysis of the cell morphology. Graphs represent the quantification of cell rounding induced by (A) TcdB or (B) TcdA over time and shows the mean percentage values of round cells (in % from total cells), calculated from triplicates (three independent wells). Error bars represent ±SD. ‘Mock’ indicates cells without dronedarone preincubation and without toxin-treatment.
